# Object-scene semantics correlation analysis for image emotion classification

**DOI:** 10.3389/fnins.2025.1657562

**Published:** 2025-09-10

**Authors:** Zibo Zhou, Zhengjun Zhai, Huimin Chen, Sheng Lu

**Affiliations:** Northwestern Polytechnical University, Xi'an, China

**Keywords:** human cognition, semantic attention, adaptive fusion, polarity-aware contrastive loss, image emotion classification

## Abstract

**Introduction:**

Image emotion classification (IEC), which predicts human emotional perception from images, is a research highlight for its wide applications. Recently, most existing methods have focused on predicting emotions by mining semantic information. However, the “affective gap” between low-level pixels and high-level emotions constrains semantic representation and degrades model performance. It has been demonstrated in psychology that emotions can be triggered by the interaction between meaningful objects and their rich surroundings within an image. Inspired by this, we propose an Object-Scene Attention Fusion Network (OSAFN) that leverages object-level concepts and scene-level reasoning as auxiliary information for enhanced emotional classification.

**Methods:**

The proposed OSAFN designs two different strategies to extract semantic information. Specifically, concepts are selected by utilizing an external concept extraction tool, and an Appraisal-based Chain-of-Thought (Appraisal-CoT) prompting is introduced to guide large language models in generating scene information. Next, two different attention-based modules are developed for aligning semantic features with visual features to enhance visual representations. Then, an adaptive fusion strategy is introduced for integrating the results of both the object-semantic stream and the scene-semantic stream. Additionally, a polarity-aware contrastive loss is proposed to model the hierarchical structure of emotions, improving the discrimination of fine-grained emotional categories.

**Results and discussion:**

To evaluate the effectiveness of OSAFN, we conducted numerical experiments on four affective datasets. The results demonstrate that OSAFN achieves superior performance and represents a notable contribution in the field of IEC.

## 1 Introduction

In recent years, with the rapid development of the Internet and the widespread adoption of mobile devices, social networks have become an integral part of modern life. Consequently, a large number of images have been generated and shared on-line by users. The need to extract valuable information from this vast volume of visual data has increased significantly. Image emotion classification (IEC), which simulates the process of human emotional perception, aims to analyze and infer the emotional semantics of images. Investigating image emotions facilitates a range of real-world applications, including decision-making (Lerner et al., 2015), mental disease treatment (Angermeyer et al., 2010), smart advertising (Sánchez-Núñez et al., 2020) etc.

In the early stages, studies in IEC revealed mainly high-level emotions from the global features of the entire images (Yang et al., 2017; He and Zhang, 2018), neglecting the fact that emotions can also be evoked by local regions. Relevant studies have attracted the extensive attention of researchers. Some studies have focused on using the object detection method to integrate object semantics to infer emotions (Zhang J. et al., 2022; Zhang et al., 2021). However, these methods often produce overlapping regions, which introduces substantial noise into the process. Other studies focused on using image saliency detection to extract local features of the image (Song et al., 2018; Fan et al., 2017; Wu et al., 2020). Since image saliency detection mainly distinguishes the differences between the local and rest parts of the image, the salient area does not necessarily represent emotion. Hence, we argue that directly mapping holistic or regional features to emotion labels may underestimate the wide affective gap between low-level pixels and high-level emotions.

Complementing these technical approaches, psychological insights provide a deeper understanding of how emotions are elicited by images. Psychologist Frijda (2009) proposed the concept of “emotional stimuli,” arguing that human emotions are shaped both by individual objects and the broader scene in affective images. Similarly, Barrett and Kensinger (2010) argued that the human brain generates emotional experiences by combining information about visual objects with contextual cues. Moreover, the work of Lindquist et al. (2015) indicated that language plays a pivotal role in the emotional cognitive process of the human brain. It acts as a “glue” between emotion and sensory experience, which reflect emotions explicitly through linguistic semantics and implicitly via logical associations. Based on these psychological insights, we argue that the incorporation of language provides a more precise and interpretable medium to capture the correlation between objects and scenes, thereby enhancing the emotion classification capability of the IEC model.

Based on the above studies, we herein propose the Object-Scene Attention Fusion Network (OSAFN), which aims to bridge the gap between visual features and emotions by leveraging the rich information encoded in language. Specifically, we utilize a large language model (LLM) to generate scene information. Taking into account the sensitivity of LLM, we design an Appraisal-based Chain-of-Thought (Appraisal-CoT) prompt, which guides the reasoning process of LLM and simulates human-like interpretation of the scene. In addition, visual-scene attention is designed to filter out irrelevant visual information and precisely locate scene-related regions by the guidance of the generated description. Then, while conventional object semantics do not have a clear correlation with emotions, we adopt emotional concepts—which are highly abstract and closely related to emotional content—as guidance for object-level feature extraction. To take advantage of meaningful concepts, we propose visual-object attention to locate visual objects and align visual objects with concepts. Last, an adaptive fusion strategy is proposed to compute the final image representation based on object-level features and scene-level features. Since the cross-entropy loss function imposes the same penalty on false samples, it produces more errors for the model. To address this issue, we design a polarity-aware contrastive loss function based on the hierarchical structure of the emotion model. This loss introduces a hierarchical penalty and enforces a hierarchy constraint during contrastive learning, helping the model to better distinguish between emotions at different levels. A large number of experiments show that our framework performs well on six public affective datasets: Flickr & Instagram (FI), Twitter I, Twitter II, and EmotionROI.

In short, the contributions of this paper are as follows:

1) We propose a novel OSAFN framework for IEC. Unlike existing methods that simply combine object and scene information, OSAFN jointly designs two semantic feature extraction modules. The object branch extracts emotional concepts, while the scene branch generates psychologically grounded descriptions with large language models. These complementary features are integrated to effectively enhance IEC performance.
2) We construct a CoT prompt to guide the LLM in generating stepwise descriptions based on the appraisal theory. The stepwise reasoning process improves the model's understanding of the emotional context within the textual information.
3) We design a polarity-aware contrastive loss that leverages the hierarchical structure of emotion labels, encouraging learned representations to preserve class-level distinctions and sentiment-level similarities in the embedding space.

The remainder of this paper is organized as follows. Section 2 reviews the existing methods of the IEC. Section 3 provides an overview of the proposed method, including the model architecture and loss functions used in the training procedure. In Section 4, conducted on public affective datasets, various experiments, including comparisons, ablation studies, and visualizations, are described. Finally, we present our conclusions and discuss future directions in Section 5.

## 2 Related work

### 2.1 Image emotion classification

To date, Image emotion classification (IEC) is not a simple task to find because observers may have a different perspective for the same image, which inevitably leads to abstracting a distinct perception. There are three methods that help researchers map low-level visual features to high-level emotions, which are traditional methods, mid-level methods, and deep learning methods.

#### 2.1.1 Traditional methods

In the early days, researchers in IEC focused mainly on utilizing manually designed approaches to extract low-level features from images, such as color and shape, to classify emotions in images. Siersdorfer et al. (2010) obtained 64-bit RGB histogram features from the global color histogram of the images, then divided the image into 16 equal blocks, extracting local color histogram features from each block for emotion detection. Lu et al. (2012) studied the emotional connotations of shape features such as roundness and angles, inferring image emotions from these low-level features. Furthermore, inspired by the influence of art, Zhao et al. (2014) designed features based on art regulation, such as balance, movement, harmony, and color classification, and used these features to train support vector machines for emotion recognition. However, human emotion cannot be triggered directly by low-level features in the images. The approach mentioned above has only good performance in some small-scale datasets, which are still difficult to establish a robust relationship between low-level features and emotional semantics.

#### 2.1.2 Mid-level methods

Since there is a huge gap between low-level features and high-level semantics, many researchers have started to build mid-level representations to express the emotion of the image. Borth et al. (2013) introduced Adjective Noun Pairs (ANPs) and proposed a visual concept detector called Sentibank. Images were fed into Sentibank to obtain visual concepts with associated detection probabilities. Ali et al. (2017) employed two pre-trained neural networks to extract object-level and scene-level semantics from images and subsequently trained linear mixture models using these semantic features to predict emotional label distributions. Similarly, Zhang et al. (2020) applied object detection techniques to identify multiple objects within each image and used Bayesian networks to model the associations among these semantic combinations and emotional states.

Compared to low-level visual features, mid-level semantic features are more interpretable to humans and exhibit stronger correlations with emotional content. However, a semantic gap remains, and existing learning frameworks have not demonstrated substantial improvements over traditional emotion recognition methods.

#### 2.1.3 Deep learning methods

Both traditional and mid-level methods perform feature extraction and train the model in separate steps. Unlike these two methods, for end-to-end approaches, features are extracted through deep learning models, with the features gradually optimized as the model is trained. You et al. (2017) combined attention mechanisms to infer emotion-related areas and correlated these local regions with descriptive emotional attributes. Unlike previous works based on global analysis, this represented the first shift in image emotion classification from a global to a local approach. In addition, He et al. (2019) proposed a multi-attention pyramid network aimed at extracting local features from feature maps of various scales and leveraging a self-attention mechanism to associate these features for emotion inference. Psychological studies have shown that image emotions are evoked by specific, meaningful content within an image. Lan et al. (2023) proposed a hyper network emotion fusion framework to model aesthetic and emotional features jointly for better prediction. Similarly, he then introduced a dynamic perception rectification algorithm to improve the balance between quality and diversity in generative models through adaptive sample reweighting (Lan et al., 2024). Besides, Zhou et al. (2025) designed an emotion-aware image captioning model that integrates visual-textual features and cross-modal attention for fine-grained emotion recognition. Based on these observations, we propose a novel deep learning method based on research in psychology to predict image emotions. Specifically, we design various semantics information to mine emotion-related regions, and fuse image features with semantic features, combined with polarity-aware contrastive loss to classify image emotions.

### 2.2 CoT prompting

A sequence of reasoning, called Chain of Thought (CoT), helps break down complex problems into manageable subtasks. Large language models (LLMs) exhibit enhanced performance and improved interpretability as a consequence. CoT prompting integrates prompt-based learning with systematic reasoning, allowing LLMs to handle abstract or multi-step problems without additional fine-tuning. Wei et al. (2022) constructed a chain of thoughts prompt with a few shots that markedly improves accuracy in arithmetic and logical tasks. Kojima et al. (2022) later demonstrated that zero-shot reasoning in LLMs can be facilitated by simple instructions like 'Let us consider step by step.' Numerous CoT prompts have been introduced in different studies, including the automated generation of CoT demonstrations (Zhang Z. et al., 2022) and the implementation of a meticulous search through multiple rationale candidates using a tree search method (Yao et al., 2023). In addition, LLMs can also address novel tasks using CoT prompting, Coconut (Hao et al., 2024) presents a Chain-of-Continuous-Thought, and CCoT (Cheng and Van Durme, 2024) uses a Compressed CoT, producing content-rich and continuous contemplation tokens.

Recently, CoT prompting has been utilized to address emotional challenges. Wu et al. (2024) designed a deconstructed reasoning framework to assist LLMs in obtaining emotion-cause pairs via stepwise inference. PEAR (Li et al., 2025) presented a CoT architecture that integrates visual and textual modalities for enhanced interpretability in sentiment reasoning. Simultaneously, current research (Li et al., 2024) improves the emotional generation capacity of LLMs by integrating an emotional CoT, allowing models to more effectively integrate affective elements during response formulation. Although most of the research remains focused on text-only tasks, these results illustrate the success of CoT prompting in emotional computing.

In light of these findings, we introduce the Appraisal-based Chain-of-Thought (Appraisal-CoT) methodology. This approach is grounded in psychological theory and directs the model through four cognitive dimensions: perception, goal, agency, and synthesis during the description generation process, which enhances the LLM's comprehension and interpretation of the emotional significance of visual scenes.

## 3 Method

**Figure 1:**
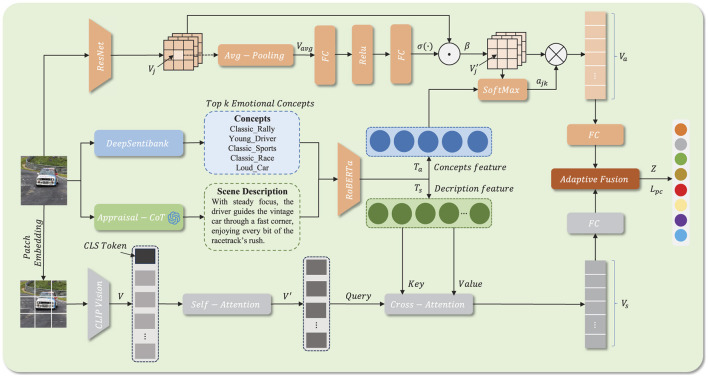
” Overview of the proposed OSAFN.

Figure 1 illustrates the complete workflow of our proposed network, which is designed with three modules: stimuli extraction, semantic attention, and adaptive fusion. First, we design an Appraisal-based Chain-of-Thought (Appraisal-CoT) prompt to guide a Large Language Model (LLM) in generating scene descriptions and employ DeepSentiBank to represent objects through emotional concepts. Next, two different attention strategies are designed to reinforce the visual feature from the object and scene semantics, respectively. Finally, the final fused representation is processed by the adaptive fusion module. In addition, the proposed polarity-aware contrastive loss function leverages the hierarchical structure inherent in emotion models to address the challenge of distinguishing between similar emotion classes.

### 3.1 Stimuli extraction

Image emotion classification (IEC) is more challenging than traditional vision tasks (Yang et al., 2018b,a; Peng et al., 2016), as emotions are abstract and ambiguous, making them difficult to infer directly from images. Since language plays a crucial role in emotion perception, we leverage an LLM to generate textual descriptions that capture scene-level semantics. Meanwhile, we use the DeepSentibank to extract emotional concepts that serve as emotion-enhanced object features.

#### 3.1.1 Object

Previous works (Zhou et al., 2017; Ahsan et al., 2017) have shown that emotion can hardly be revealed at the object level. Unlike these methods, the emotional concept from DeepSentibank (Chen et al., 2014) provides us cues to reconsider the mining of object semantics. Initially, Borth et al. (2013) used the 24 emotions from Plutchik's model (Plutchik, 1980) to search Flickr and YouTube, collecting about 316,000 images and videos. They then extracted adjective–noun pairs (ANPs) from the associated text tags, retaining 3,244 concepts such as “beautiful flowers” and “sad eyes” after filtering. Then, they retrieved images for each ANP and removed those with too few samples, creating the VSO dataset containing 1,553 ANPs and corresponding images. Building on this, Chen et al. collected 2,089 ANPs and 867,919 images to construct a new dataset for training DeepSentibank. Its final fully connected layer contains 2,089 neurons, each representing one ANP. Given an input image, DeepSentibank outputs the probability distribution across all 2,089 concepts, providing mid-level emotional representations that allow emotion inference without directly viewing the image. For each emotion concept, feature extraction is carried out using the RoBERTa model (Liu et al., 2019), which is based on the BERT model language masking method (Devlin et al., 2019), and trains the system to predict purposely hidden content within otherwise unannotated language instances. The object-level features $T_{a}=\left\{t_{1},\ldots ,t_{k},\ldots ,t_{l}\right\}\in {\mathbb{R}}^{l\times d_{t}}$ are obtained, where *l* is the number of emotion concepts that we select, *d*_*t*_ is the embedding dimension, and *t*_*k*_ represents the features of the concept *k*.

#### 3.1.2 Scene

**Figure 2:**
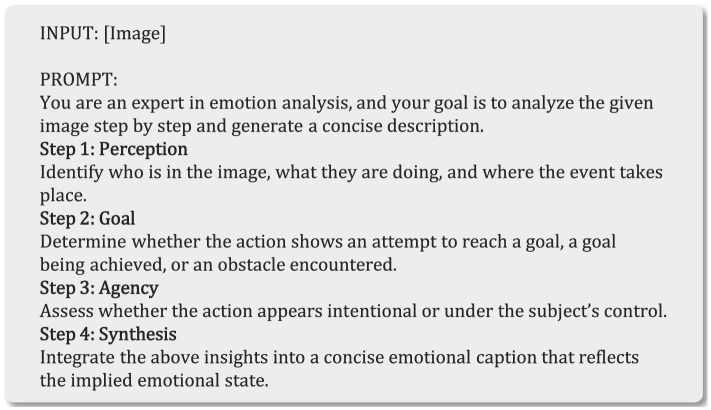
A template of an Appraisal-CoT prompt.

When viewing an image, an observer typically reconstructs the depicted scene by simulating contextual events and interpreting human actions. Inspired by this cognitive process, we incorporate both the event details and the human behaviors as essential components of the scene-level context. However, descriptions generated by conventional VQA models (Liu et al., 2023b) often lack sufficient contextual depth and nuanced semantic understanding. We argue that such flat descriptions are insufficient to capture the implicit emotional states conveyed in complex scenes. Fortunately, appraisal theory (Lazarus, 1991) provides a psychological perspective on how people generate emotions: emotions arise not only from external events but also from individuals' evaluations of the personal relevance, significance, and degree of perceived control over its outcome of an event. Based on this principle, we propose Appraisal-CoT, a chain-of-thought prompting strategy that guides a large language model (LLM) through step-by-step cognitive evaluations to produce richer and more accurate descriptions of the emotional landscape within an image. The overall Appraisal-CoT of this is illustrated in Figure 2. Similarly to the concept encoding process, we denote the scene description as *X*, the feature is calculated as follows:


(1) $$\begin{array}{l} T_{s}=RoBERTa\left(E\right)\in {\mathbb{R}}^{e\times d_{t}} \end{array}$$

where


(2) $$\begin{array}{l} E=Embedding\left(Tokenizer\left(X\right)\right) \end{array}$$

is Roberta's tokenizer that encodes description into a fixed-length sequence. *e* is the maximum length of the sequence and *d*_*t*_ is the embedding dimension. The output *T*_*s*_ represents the final scene-level features.

### 3.2 Semantic attention

#### 3.2.1 Visual-object attention

While the concepts from DeepSentibank capture emotion-related content to some extent, more informative and visually grounded semantics are needed to guide attention and enhance feature complementarity. In practice, for each image, the pre-trained ResNet50 (Deng et al., 2009) is used to capture local details and salient regions, the extract visual features $V=\left\{v_{1},\ldots ,v_{j},\ldots ,v_{m}\right\}\in {\mathbb{R}}^{m\times d_{v}}$ is obtained, where *m* is the number of regions, *d*_*v*_ is the feature dimension of each region, and *v*_*j*_ represents the *j*-th region of local features in the feature map. We first apply global average pooling to the visual feature map *V*:


(3) $$\begin{array}{l} v_{avg}=\frac{1}{m}\overset{m}{\underset{j=1}{\sum }}v_{j} \end{array}$$

Then, we pass the pooled features through a two-layer fully connected network to explore which visual regions are most relevant to emotion expression:


(4) $$\begin{array}{l} \beta =\sigma \left(W_{1}\left(Relu\left(W_{2}v_{avg}\right)\right)\right) \end{array}$$

where $W_{1}\in {\mathbb{R}}^{m\times r}$ and $W_{2}\in {\mathbb{R}}^{r\times d_{v}}$ represent parameter matrices, *r* is the reduction ratio that controls the dimension of the hidden layer in the attention computation. Last, each patch vector in $v_{j}\in {\mathbb{R}}^{m\times d_{v}}$ is multiplied by its corresponding weight β_*j*_ ∈ [0, 1], producing the enhanced visual features:


(5) $$\begin{array}{l} v_{j}^{\prime }=\beta \cdot v_{j} \end{array}$$

Our goal is to automatically find this kind of correlations between emotional concepts and image regions. Thus, multi-head attention is used to locate emotion-related regions for each emotion concept, we compute attention weights for each concepts, where the softmax function normalizes the importance scores and ensures a balanced contribution between modalities. Specifically, two low-rank projection matrices are used to project the two feature vectors (i.e., visual features $v_{j}^{\prime }$ and concept features *t*_*j*_) into a *d*-dimensional common space. Then they are fused with element-wise multiplication:


(6) $$\begin{array}{l} \alpha_{jk}=\text{softmax}\left(\frac{v_{j}^{\prime }W_{v}\left(t_{k}U_{k}\right)}{\sqrt{d}}\right) \end{array}$$

where $W_{v}\in {\mathbb{R}}^{d_{v}\times d}$ and $U_{k}\in {\mathbb{R}}^{d_{t}\times d}$ represent parameter matrices. $\sqrt{d}$ denotes the scaling factor. Then, for each visual feature, we calculate the weighted average of all visual features for the word *t*_*k*_ using the attention score α_*jk*_ as follows:


(7) $$\begin{array}{l} V_{a}=\overset{m}{\underset{j=1}{\sum }}\alpha_{jk}\cdot t_{k} \end{array}$$

where $V_{a}\in {\mathbb{R}}^{m\times d_{v}}$ represents the object-level feature.

#### 3.2.2 Visual-scene attention

Our goal is to add scene semantic into visual features, which obtain non-explicit abstract information from the scene description. Unlike emotional concepts that are relatively independent, scene descriptions often include interrelated details about actions and events, which makes it essential to capture their internal dependencies. Therefore, for each image, the visual encoder of the pre-trained CLIP (Radford et al., 2021) is used to capture strong contextual relationships, the extracted visual feature $V=\left\{v_{cls},v_{1},\ldots ,v_{k},\ldots ,v_{n}\right\}\in {\mathbb{R}}^{\left(n+1\right)\times d_{v}}$ is obtained, where *n* is the number of patches, *d*_*v*_ is the feature dimension of each patch, and *v*_*k*_ represents the *k*-th patch feature in the feature map. First, we use a self-attention mechanism to help the model understand how different parts of the image relate to each other, the equation is as follows:


(8) $$\begin{array}{l} V^{\prime }=Attention^{self}\left(WV\right) \end{array}$$

Where *W* is is the learnable matrix. Next, we apply cross-attention to model the interaction between the text and the image. Here, we regard enhanced visual features *V*′ as query, and scene features *T*_*s*_ as key and value. The equation is as follows:


(9) $$\begin{array}{l} V_{s}=Attention^{cross}\left(V^{\prime },T_{s},T_{s}\right) \end{array}$$

where $V_{s}\in {\mathbb{R}}^{m\times d_{v}}$ represent scene-level feature.

### 3.3 Adaptive fusion

Through the above steps, object-level and scene-level features can be obtained. However, as many images on social networks may lack notable objects and instead depend on the context of the scene to express emotions, we feed two features into the adaptive fusion module for effective fusion. Due to the different contributions of object-level and scene-level features, the proposed adaptive fusion module is designed to capture the discriminative information of the modalities features by adaptively tuning the balanced weight instead of setting up a fixed weight. Specifically, the final representation *Z* of fused features is assembled by:


(10) $$\begin{array}{l} Z=\mu_{k}V_{a}+\left(1-\mu_{k}\right)V_{s} \end{array}$$

where the adaptive balanced weight μ_*k*_ ∈ (0, 1) is computed by:


(11) $$\begin{array}{l} \mu_{k}=\sigma \left(V_{a}W_{z}+V_{s}U_{z}\right) \end{array}$$

where the parameter matrices *U*_*z*_ and $U_{z}\in {\mathbb{R}}^{d\times d}$, and they are both learned through the learning process. In addition, σ(·) is the sigmoid function.

### 3.4 Polarity-aware contrastive loss

**Figure 3:**
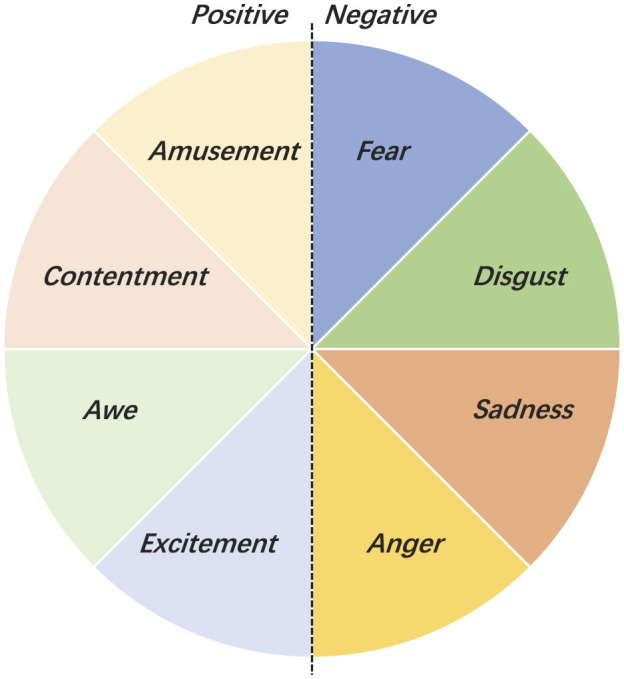
Mikel's emotion wheel.

Psychological theory (Mikels et al., 2005) indicates that emotion labels are organized hierarchically. For example, Mikel's emotion wheel (Ekman, 1992) is shown in Figure 3, amusement, contentment, awe, and excitement fall into positive emotions, while fear, anger, disgust, and sadness fall into negative emotions. We observe that emotion categories with the same polarity tend to exhibit higher semantic similarity, making them harder to distinguish than those with opposite polarities. To address this issue, we propose a polarity-aware contrastive loss that uses the hierarchical structure of labels to better category the sample space.

Specifically, at the class level, a positive sample is randomly selected from the same emotion class as the anchor within the batch, while a negative sample is drawn from a different category that shares the same emotion polarity as the anchor. The class-level contrastive loss is defined as:


(12) $$\begin{array}{l} L_{cls}=\underset{i\in I}{\sum }\frac{-1}{\left|P_{cls}\left(i\right)\right|}\underset{p\in P_{cls}\left(i\right)}{\sum }\log\frac{\exp\left({\text{z}}_{i}\cdot {\text{z}}_{p}/\tau \right)}{\sum_{a\in A_{i}}\exp\left({\text{z}}_{i}\cdot {\text{z}}_{a}/\tau \right)} \end{array}$$

where *I* denotes the set of anchor indices within a mini-batch, and $Z_{i}\in {\mathbb{R}}^{d}$ represents the feature embedding of the *i*-th anchor sample. *P*_*cls*_(*i*) is the set of class-level positive samples that share the same emotion category as the anchor. *A*_*i*_ is the set of all candidate samples used for contrastive comparison with anchor *i*, typically composed of all other samples in the batch excluding the anchor itself, and τ is a temperature hyperparameter controlling the sharpness of the softmax distribution.

At the polarity level, the positive sample is selected from a different emotion category that shares the same sentiment polarity as the anchor, while the negative sample is drawn from a category with the opposite polarity. The polarity-level contrastive loss is defined as:


(13) $$\begin{array}{l} L_{pol}=\underset{i\in I}{\sum }\frac{-1}{\left|P_{pol}\left(i\right)\right|}\underset{p\in P_{pol}\left(i\right)}{\sum }\log\frac{\exp\left({\text{z}}_{i}\cdot {\text{z}}_{p}/\tau \right)}{\sum_{a\in A_{i}}\exp\left({\text{z}}_{i}\cdot {\text{z}}_{a}/\tau \right)} \end{array}$$

where *P*_*pol*_(*i*) is the set of polarity-level positive samples that share the same sentiment polarity with the anchor but belong to different emotion categories.

We combine the class-level and polarity-level losses into a multi-level hierarchical contrastive loss:


(14) $$\begin{array}{l} L_{Con}=\frac{1}{2}\left(L_{cls}+L_{pol}\right) \end{array}$$

Finally, we add the standard cross-entropy loss *L*_*CE*_ to form the complete objective:


(15) $$\begin{array}{l} L=L_{Con}+L_{CE} \end{array}$$

## 4 Experiment

### 4.1 Dataset

Affective datasets for experiments are collected from social media, including Twitter I (You et al., 2015), Twitter II (Borth et al., 2013), EmotionROI (Peng et al., 2015), FI (You et al., 2016), Details of the datasets are shown in Table I.

**Table 1:** Statistic of the involved image emotions datasets.

**Dataset**	**Sum**	**Positive**	**Negative**	**Classes**
Twitter I	1269	769	500	2
Twitter II	603	470	133	2
Flickr & Instagram	23308	16430	6878	8
EmotionROI	1980	660	1320	6

Twitter I contains 1,269 images labeled with positive or negative emotions based on the two-polarity model. This dataset is built on a binary emotion model, where each image is annotated by five AMT participants. Each participant selects one emotion label (positive or negative), and the label receiving at least three votes is assigned as the emotion annotation for the image.

Like the Twitter I dataset, Twitter II was annotated using the AMT approach with three rounds of labeling: text-based, image-based, and text–image-based. In each round, three AMT participants were invited, and an image was labeled as “agreed” if at least two participants selected the same label. Through this process, the dataset retained 470 positive images and 133 negative images.

The images of EmotionROI are collected from Flickr by searching the six categories that are Ekman's 6 basic emotions (anger, disgust, joy, fear, sadness, and surprise). In addition to providing emotion distribution labels, the dataset also includes valence–arousal (VA) scores. To ensure that the selected images evoke emotions through low-level visual features, Peng et al. removed images containing obvious facial expressions or explicit emotional text from the dataset. One thousand nine hundred and eighty images are finally assembled in total, of which 330 images were collected in each emotion category. As with the FI dataset, the different splits of the EmotionROI dataset are labeled as EmotionROI 6 and EmotionROI 2, respectively.

The Flickr & Instagram (FI) dataset is collected from Flickr and Instagram based on the Mikels emotion model. first collected three million weakly labeled emotional images from Flickr and Instagram, then screened and filtered them based on label consistency and class balance. Images in the FI dataset were annotated by Amazon Mechanical Turk (AMT) participants, with each image assigned to five workers. Each worker selected one emotion from eight categories, and a label was assigned to the image only if at least three workers agreed on the same category. Due to its large volume and relatively low label noise, the FI dataset is one of the most used datasets for emotion classification tasks. The different split types of FI are marked as FI 8 and FI 2, respectively.

### 4.2 Training setup

The entire network is optimized from end to end in the affective datasets using the polarity-sensitive contrastive loss function proposed in the previous section. Following previous work (Zhou et al., 2025), FI and EmotionROI are split into 80% training, 5% validation, and 15% testing, while Twitter I and II are split into 80% training and 20% testing. In addition, data augmentation including random rotation, gaussian blur, and color enhancement are used during the training process. All images are resized to 224 × 224. To preserve the main image information during data augmentation, 10% of the image width is randomly cropped from the left or right side if the aspect ratio is greater than 1, while 10% of the image height is randomly cropped from the top or bottom side if the aspect ratio is less than 1. The backbone of OSAFN is the ViT-B / 32 encoder pre-trained on the COCO (Lin et al., 2014) dataset and ResNet50 pre-trained on the ImageNet dataset. In the object branch, the feature map from the last convolutional layer is used, resulting in 49 feature vectors of dimension 2048 for each image.

Based on extensive observations and prior studies, we found that the top five emotional concepts detected by DeepSentibank provide a more accurate and expressive description of image content. Accordingly, we selected the top five adjective-noun pairs for each image to serve as emotional concepts. For scene-level semantics, we employ GPT-4o guided by our Appraisal-CoT prompting strategy to generate descriptive captions. To ensure consistency in feature dimensions across modalities, all semantic information, including emotional concepts and scene descriptions, is encoded using the same RoBERTa-base model.

We train the model using the AdamW optimizer with a batch size of 64 for 50 epochs. The learning rate starts at 1e-4, with linear warm-up for the first 5 epochs and decay by 0.2 every 5 epochs. The weight decay is set to 1e-2. All experiments were performed using PyTorch on two NVIDIA RTX 4090D GPUs with 48GB of CPU memory.

### 4.3 Comparison

To validate the effectiveness of our proposed framework, we compare it with a series of state-of-the-art methods. The experiments are carried out in four benchmark settings, including FI (8-class and 2-class), EmotionROI (6-class and 2-class), Twitter I, and Twitter II. Detailed information on these methods is introduced as follows:

1) SOLVER (Yang et al., 2021) built an emotion graph to extract emotional relationships between different objects and explored the correlation between objects and the scene for emotion classification.
2) Yang et al. (2023) explored the relationship between concept and emotion using a knowledge graph, and proposed a multi-task learning deep model to improve emotion recognition.
3) MDAN (Xu et al., 2022) proposed a two-branch architecture to leverage emotion hierarchy and the correlation between different affective levels and semantic levels. It also introduced a novel emotion category model based on psychological knowledge.
4) SimEmotion (Deng et al., 2022) was a language-supervised model that effectively leveraged the rich semantics of the image and text of CLIP, which combines the multi-modal features to drive the model to gain stronger emotional discernment with language prompts.
5) EERCA-ViT (Wang et al., 2023) enhanced ViT-based emotion recognition by combining region-level excitation and context-aware learning through a two-branch architecture with specialized attention modules.
6) CMANet (Yang et al., 2024) was designed to utilize concepts to extract visual and semantic features, respectively. Two fusion strategies are applied to achieve complementation between visual and semantic features.
7) MASANet (Cen et al., 2024) was dedicated to integrating specific uni-modal tasks for multi-modal joint emotion analysis by introducing prior knowledge from textual domains.
8) VSCNet (Zhang H. et al., 2024) combined spatial attention with an affective region discovery branch to enhance emotional feature extraction.
9) OEAN (Zhang J. et al., 2024) combined object-guided visual attention and semantic modeling of object-emotion mappings, and fused both modalities via a BiGRU to improve image emotion classification.

Comparisons between OSAFN and baseline methods are presented in Table 2. It can be seen that our method achieved comparable experimental results. Methods such as SOLVER and OEAN focus on utilizing detected objects to mine emotional region directly. Although object-level features help capture fine details, they may fail when emotions come from a scene rather than a specific object. In contrast, our model combines both object and scene understanding, achieving stronger performance in such settings.

**Table 2:** A comparison with state-of-the-art methods.

**Method**	**FI(8)**	**FI(2)**	**EROI(6)**	**EROI(2)**	**Twitter I**	**Twitter II**
Yang et al.	65.46	-	55.38	-	-	-
EERCA-ViT	71.18	92.54	66.50	89.39	-	-
SOLVER	72.34	-	62.12	-	85.27	83.19
VSCNet	73.04	-	62.86	-	85.32	-
OEAN	73.40	-	64.12	91.92	92.86	90.08
CMAnet	73.51	89.86	62.24	83.38	85.95	-
MDAN	76.35	91.01	60.66	83.96	-	-
MASANet	79.16	95.07	73.23	91.75	92.16	88.70
SimEmotion	80.33	95.42	70.54	90.40	89.76	84.21
Ours	**80.96**	**95.51**	**73.59**	**92.81**	**92.89**	**90.16**

Results are reported as classification accuracy (%) in four affective datasets. EROI represents EmotionROI dataset in the table. The best results are marked in bold, and the second-best results are marked with underscores.

Next, both CMANet and Yang's method leverage emotional concepts to mine emotional regions. However, these methods overlook the emotion that may arise from the interaction between visual and semantic features. By considering abstract emotional concepts and scene descriptions in images, our method enables a more comprehensive learning of semantic information, leading to better results.

Then, we also compare with semantic-enhanced methods, including EERCA-ViT, MDAN, and VSCNet, which focus on refining visual features through multimodal feature fusion. However, these methods often lack explicit modeling of the affective reasoning process. EERCA-ViT applies dual-branch attention to filter contextual noise but fails to incorporate high-level semantic interpretation. MDAN and VSCNet integrate image and text features, but the relationship between emotion and features is unclear. Compared to these methods, our method not only enhances visual representations but also enriches the semantic representation by introducing multi-level textual information based on psychological theory, which makes the result achieve superior performance in four datasets.

In addition, MASANet and SimEmotion introduce a textual description to guide emotional understanding of the model. However, these methods are based on designed or fixed templates, which limits their flexibility and generalization. In contrast, we design a psychologically grounded CoT prompt, which encourages the model to generate image description toward an emulation of human-like contemplation. This guided reasoning helps our model capture deeper emotional meaning, As a result, with a wealth of language knowledge, we achieve a better performance (improved by 0.63%) than SimEmotion.

Above all, the proposed OSAFN consistently outperforms the state-of-the-art methods on four visual emotion datasets, demonstrating the effectiveness and robustness of our method.

### 4.4 Ablation

In this section, we conduct several experiments to verify the effectiveness of Appraisal-CoT, visual-object attention, visual-scene attention, adaptive fusion, and polarity-aware contrastive loss, respectively.

#### 4.4.1 Effects of Appraisal-CoT

**Figure 4:**
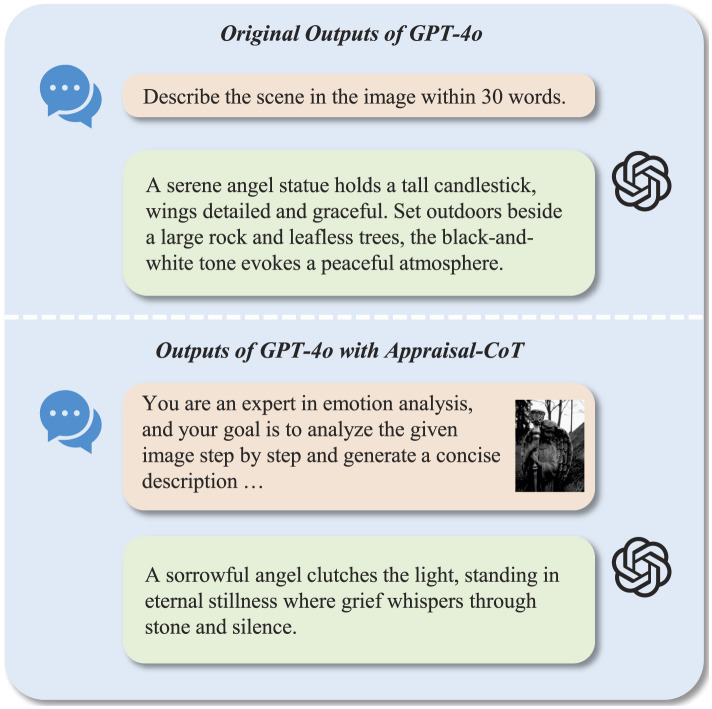
A template of Appraisal-CoT prompt.

To verify the contribution of Appraisal-CoT, we performed an ablation study on three vision-language models: GPT-4o (OpenAI, 2023), Qwen-VL (Bai et al., 2023), and LLaVA-1.6 (Liu et al., 2023a) For each model, we compare the results generated with standard captioning prompts (e.g., Describe the scene in the image) and our proposed Appraisal-CoT prompt, which incorporates step-by-step reasoning based on cognitive emotion theory. As shown in Table 3, the Appraisal-CoT significantly improves emotional understanding by guiding structured cognitive reasoning. Furthermore, Figure 4 further illustrates two captions from GPT-4o under different prompt settings. Captions generated using standard prompts often include redundant and emotion-irrelevant details (e.g., “tall candlestick,” “large rock”). In contrast, our generative captions made full use of emotional attributes and achieved better performance.

**Table 3:** A comparison of results utilizing different prompt.

**Model**	**Standard captioning**	**Appraisal-CoT**
GPT-4o	78.21	80.96
Qwen-VL	77.34	80.88
LLaVA-1.6	75.98	80.63

Results are reported as classification accuracy (%) on FI datasets.

#### 4.4.2 Effects of visual encoder

Table 4 presents the performance of our model with different visual encoders. The results clearly show that models equipped with ViT-based backbones surpass those using CNN-based frameworks, indicating that our model can effectively adapt to various visual backbones. Beyond the benefits of attention mechanisms in capturing scene information, future IEC tasks may further benefit from exploring alternative architectures or enhancements to achieve greater language-supervised improvements.

**Table 4:** A comparison of results utilizing different vision encoders.

**Visual encoder**	**FI(8)**	**FI(2)**	**EROI(6)**	**EROI(2)**	**Twitter I**	**Twitter II**
ResNet50	79.04	93.04	71.71	88.14	89.28	87.41
ViT-B/16	80.31	94.84	73.02	91.92	90.86	89.05
ViT-L/14	83.91	96.77	75.27	94.19	93.22	90.94
ViT-L/14@336	84.13	96.88	76.77	94.95	92.86	90.92
Ours	**80.96**	**95.51**	**73.59**	**92.81**	**92.89**	**90.16**

#### 4.4.3 Effects of individual branches

To further explore the effectiveness of each branch in our proposed network, we individually evaluate the classification performance of the object-level branch and the scene-level branch and compare them with the full model using the adaptive fusion module. The results are shown in Table 5. It can be observed that the using scene description solely achieves slightly higher accuracy than emotional concepts, which demonstrates scene-level semantics generated by the CoT-guided description exhibit a significant correlation with emotions. The object-only branch also performs reasonably well, capturing affective cues from emotional concepts. The proposed adaptive fusion method combines both branches and achieves the best performance across all datasets.

**Table 5:** A comparison of single-branch and fused-branch performance.

**Method**	**FI(8)**	**FI(2)**	**EROI(6)**	**EROI(2)**	**Twitter I**	**Twitter II**
Object only	77.64	92.13	70.02	89.31	90.84	87.71
Scene only	78.59	93.15	71.78	90.52	91.21	88.24
Ours	**80.96**	**95.51**	**73.59**	**92.81**	**92.89**	**90.16**

#### 4.4.4 Effects of adaptive fusion

**Figure 5:**
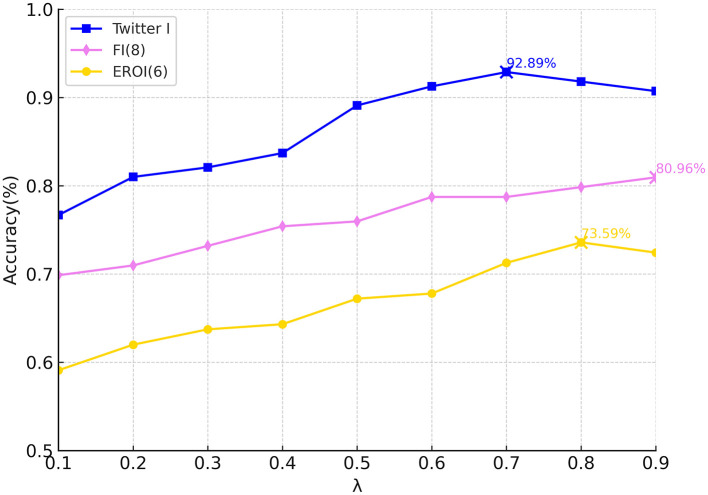
Accuracies with different values of λ in weighted summation.

Previous works often fused features using simple concatenation or average summation. To conduct a more thorough study, we first replace the proposed adaptive fusion with a weighted summation of *V*_*a*_ and *V*_*s*_, which is defined as:


(16) $$\begin{array}{c} Z=\lambda V_{a}+\left(1-\lambda \right)V_{s} \end{array}$$

where λ ranges from 0.1 to 0.9 to ensure that *V*_*a*_ and *V*_*s*_ contribute simultaneously. The experimental results with different λ values are shown in Figure 5. The highest accuracy is achieved with λ = 0.9 on FI (class 8), λ = 0.8 on EmotionROI (class 6), and λ = 0.7 on Twitter I. It can be seen that larger values of λ generally lead to better results, with the FI dataset benefiting more from higher λ values compared to the other datasets. This suggests that object features play a crucial role in classification, particularly for the FI dataset. However, when λ approaches 0.9, performance drops for EmotionROI and Twitter I due to the reduced semantic contribution from the scene features. We also evaluate concatenation fusion. As shown in Table 6, since the contribution of each feature may vary, using a fixed weight in summation or direct concatenation ignores this variation, leading to suboptimal results. Moreover, λ in the weighted summation is manually set and remains constant for all images, which explains why even the best weighted summation scores are lower than those of adaptive fusion. Overall, weighted summation performs slightly better than concatenation.

**Table 6:** A comparison of results utilizing different fusion strategies.

**Method**	**FI(8)**	**FI(2)**	**EROI(6)**	**EROI(2)**	**Twitter I**	**Twitter II**
Weighted summation fusion	78.98	93.98	71.49	90.12	90.37	88.34
Concatenation fusion	77.36	93.01	70.25	89.57	89.78	87.59
Ours	**80.96**	**95.51**	**73.59**	**92.81**	**92.89**	**90.16**

#### 4.4.5 Effects of polarity-aware contrastive loss

To validate the effectiveness of our proposed polarity-aware contrastive loss, we perform comparative experiments by replacing it with two widely used loss functions: focal loss (Lin et al., 2017) and center loss (Wen et al., 2016). The results are summarized in Table 7. Specifically, focal loss is combined with standard cross-entropy to address class imbalance by down-weighting easy samples and emphasizing hard samples during training. Center loss, on the other hand, is introduced to enhance intra-class compactness by pulling samples of the same class closer to their corresponding class centers in the feature space. Although both focal loss and center loss offer certain improvements in feature discrimination, their performance remains inferior to that of our polarity-aware contrastive loss, achieving only 78.7% and 79.2% in the FI(8) dataset, respectively. This gap highlights the advantage of explicitly modeling the hierarchical structure of emotion categories, as our loss function does, to guide the learning process in emotion classification tasks.

**Table 7:** A comparison of results utilizing different loss functions.

**Method**	**FI(8)**	**FI(2)**	**EROI(6)**	**EROI(2)**	**Twitter I**	**Twitter II**
Focal Loss + CE	78.78	93.68	71.88	91.04	89.62	87.97
Center Loss + CE	79.21	93.41	72.11	90.72	89.98	88.12
Ours	**80.96**	**95.51**	**73.59**	**92.81**	**92.89**	**90.16**

**Figure 6:**
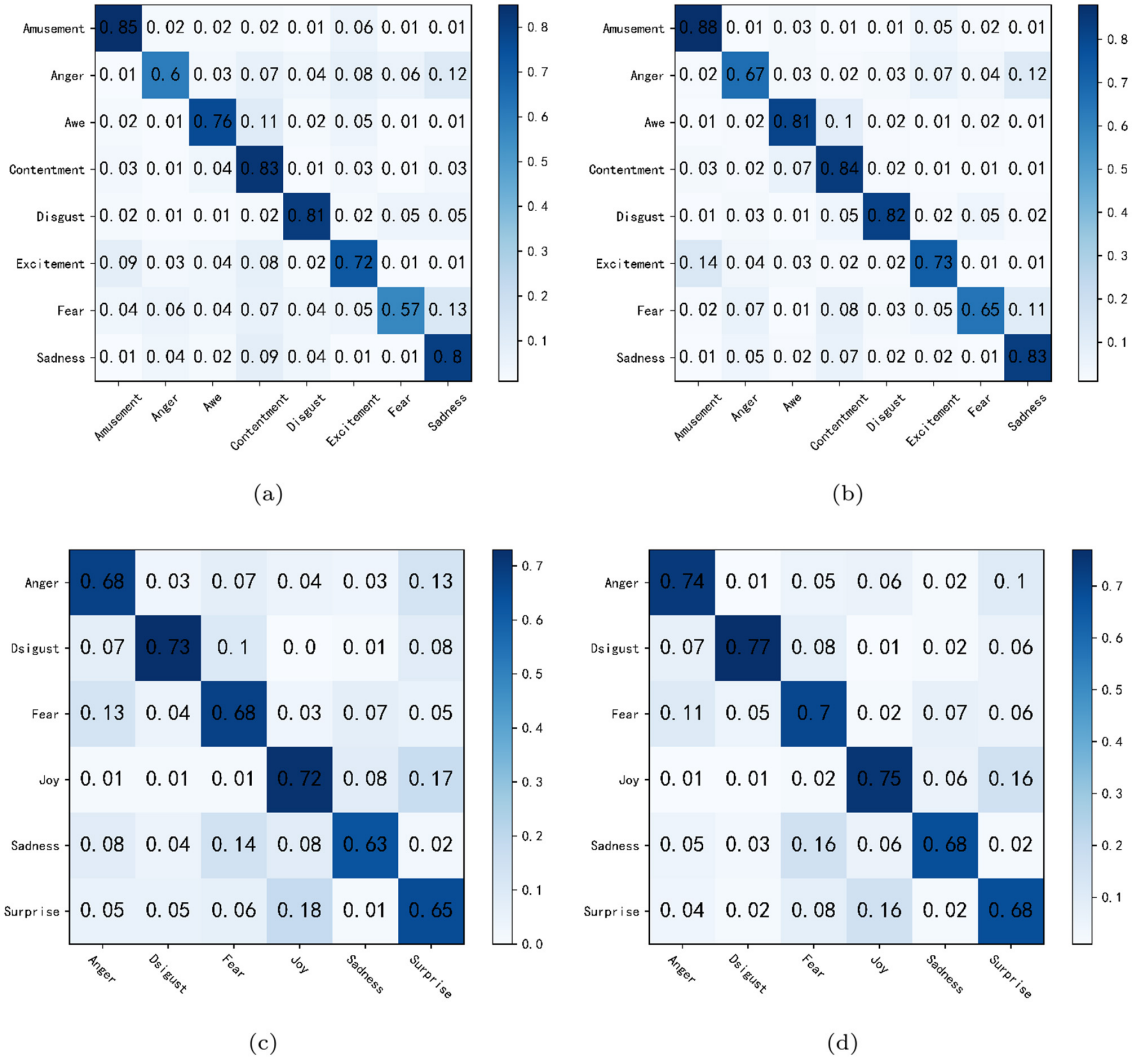
Confusion matrices for classification results from OSAFN applied to each dataset. **(a, b)** display results using cross-entropy loss and our proposed loss function with the FI dataset, respectively. Similarly, **(c, d)** illustrate the corresponding results for the EmotionROI dataset.

To further illustrate the effectiveness of the proposed polarity-aware contrastive loss *L*_*pc*_, we also visualize the precision on each emotion via confusion matrices, where Figure 6a shows the results with the cross-entropy loss and Figure 6b shows the results with our proposed loss on the FI dataset. As depicted in Figure 6a, categories such as Amusement vs. Excitement, Anger vs. Sadness, and Awe vs. Contentment are prone to misclassifications. By applying our polarity-aware contrastive loss, Figure 6b indicates that the accuracy for Amusement improves from 85% to 88%, while the confusion between Amusement and Excitement is reduced from 6% to 5%. Similarly, improvements can be observed for other categories, such as Awe and Contentment. These results confirm that our loss function penalizes frequent confusions more strongly. In addition, Figures 6c, d show the corresponding results on the EmotionROI dataset. In Figure 6c, significant confusions are observed between Anger and Surprise, as well as between Joy and Surprise. In contrast, with the use of *L*_*pc*_ (Figure 6d), the confusion between Anger and Surprise drops from 13% to 10%, while the classification accuracies for Disgust, Fear, and Joy improve from 73% to 77%, 68% to 70%, and 72% to 75%, respectively. This improvement is attributed to *L*_*pc*_'s mechanism of adaptively down-weighting well-classified samples and amplifying penalties on misclassified ones, effectively mimicking a human-like learning focus on hard samples. Last, it can be seen that the accuracy for anger and fear remains lower than that of other emotions. This is not primarily due to the model itself, but to the intrinsic features of the FI and EmotionROI datasets, where visual features of anger and fear are highly similar and result in overlapping feature distributions. With the integration of semantic features and the proposed loss function, we observe performance gains for these two categories compared with using visual features alone, indicating that our method alleviates category confusion and achieves more robust classification performance.

### 4.5 Visualization

The effectiveness of the proposed network has been rigorously evaluated through a series of comprehensive comparative analyzes and detailed ablation studies. As we are motivated by the psychological evidence that emotions are evoked by specific, meaningful content within an image, in this section, we try to intuitively illustrate the effect of the visual attention mechanism, we visualize the attention scores of concepts via heatmap in Table 8. Red denotes the visual features of the local region that have higher correlation with the concept. It can be seen that the heatmap of the relevant concept covers the corresponding region exactly, which illustrates that the proposed visual attention approach can achieve the location of the weakly supervised emotional region. Taking the last image as an example, the “group of people” represents an object, “carnival” denotes human activity, “lively and energetic” describes a scene attribute, and “laughing and smiling” infers a facial expression, suggesting the heatmap of relevant regions very accurately covers the corresponding emotional attributes. Furthermore, the attention maps for scene descriptions are shown in Table 9, where red denotes the local scene regions with higher correlation to the corresponding scene description generated by GPT-4o. It can be observed that the highlighted regions in the heatmaps align well with the contextual information, demonstrating that the proposed Appraisal-Cot prompting can effectively locate relevant emotional regions, which complements the information from emotional concepts and provides deeper insight into scene-level visual information. In addition, to provide a more intuitive explanation of the proposed method, Table 10 presents examples of emotional concepts and scene descriptions for several images. As shown in the first and second images, the ANPs are highly abstract, closely aligned with visual content, and more effective in expressing emotions. In the last image, due to the limited variety of ANPs and the constraints imposed by the emotional concept dataset, certain emotional concepts, such as “Little_Boy” and “Playful_Kids,” do not fully align with the actual image content, which affects their descriptive accuracy. In contrast, scene descriptions can better connect to the emotional context. These examples demonstrate the complementary nature of the two types of description.

**Table 8:** Attention maps corresponding to different emotional concepts.

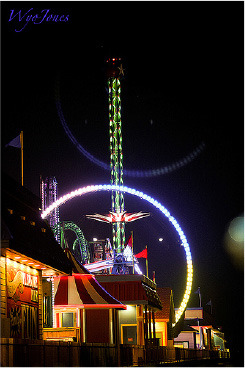	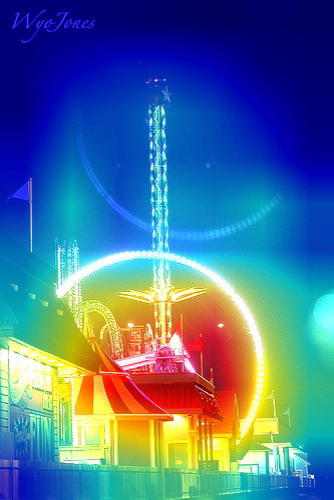	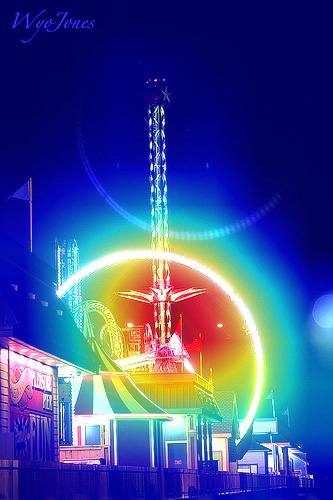	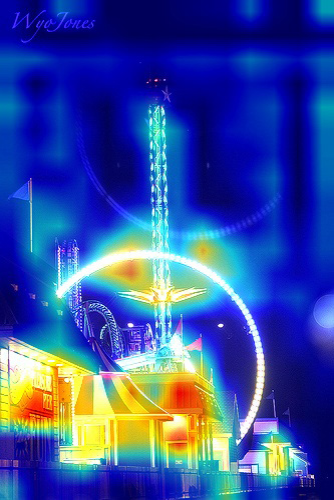	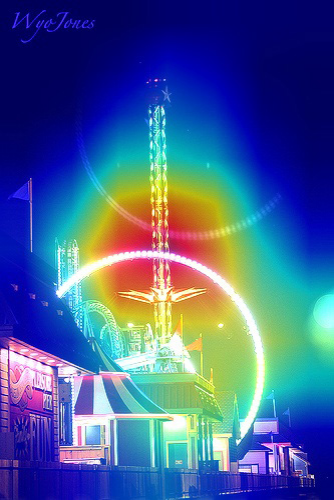	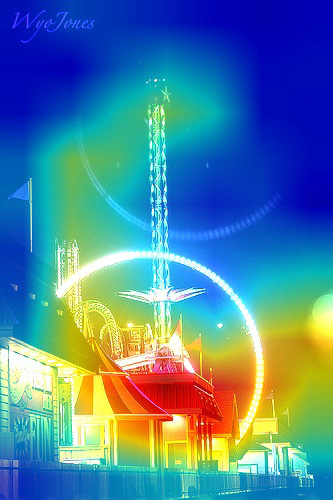
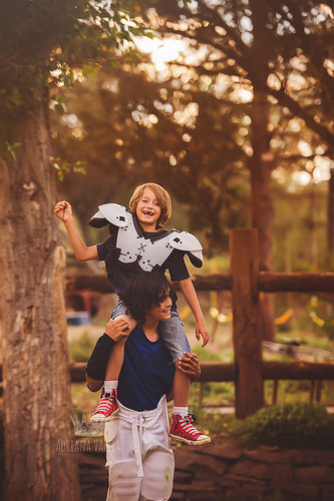	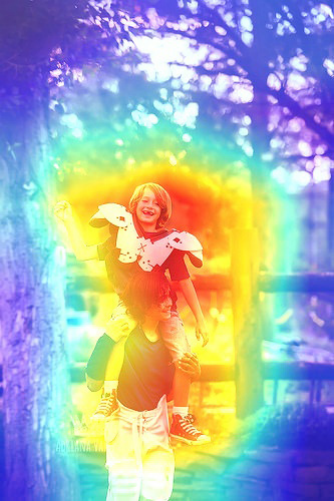	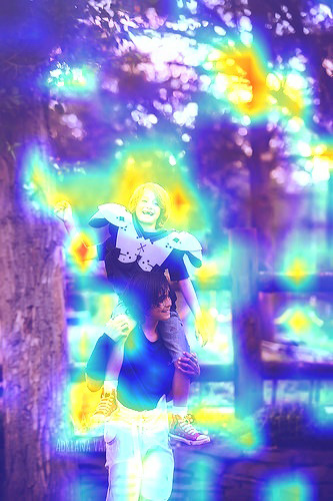	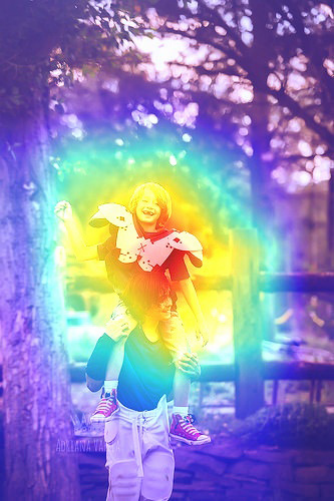	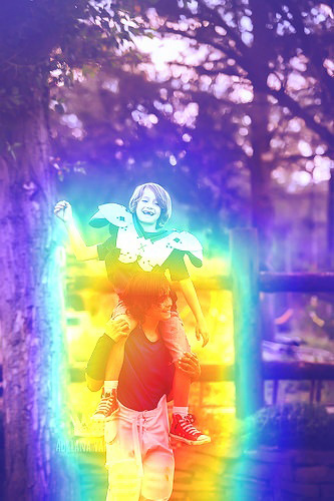	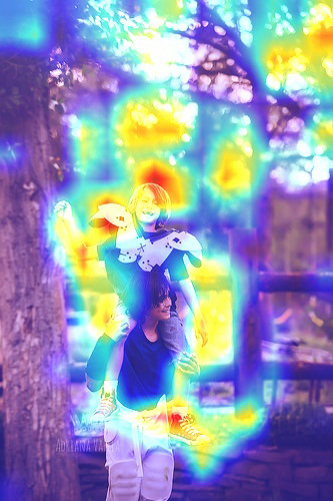
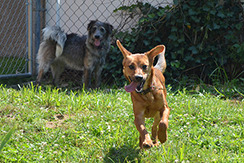	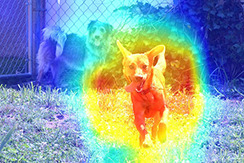	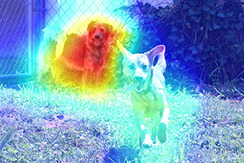	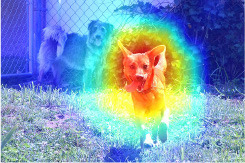	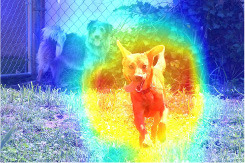	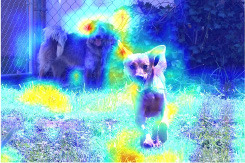

Images reprinted with permission from “Building a Large Scale Dataset for Image Emotion Recognition: The Fine Print and The Benchmark” by Quanzeng You, Jiebo Luo, Hailin Jin, and Jianchao Yang, licensed under ©2016 Association for the Advancement of Artificial Intelligence (AAAI), [Source: AAAI Conference Proceedings].

**Table 9:** Attention maps corresponding to scene descriptions.

**Image**	**Heatmap**	**Description**
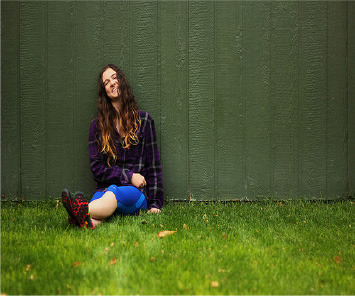	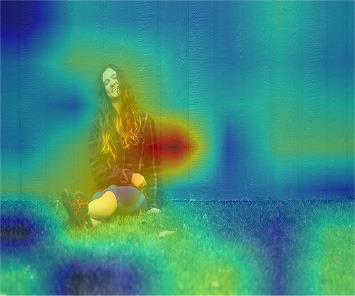	A young woman sits relaxed against a green wall, smiling peacefully, radiating calmness and contentment, as natural surroundings highlight her joyful and serene.
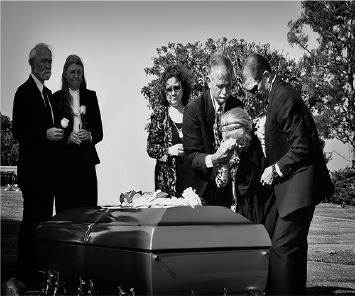	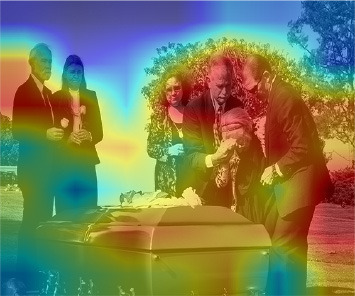	Mourners gather solemnly around a casket, expressions heavy with grief, loss, and sorrow, as comforting gestures reveal the deep emotional pain and shared mourning of a funeral ceremony.
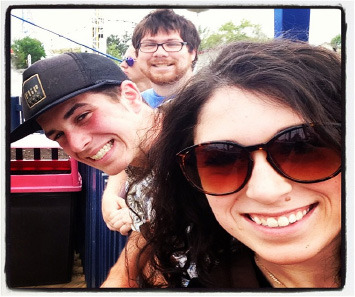	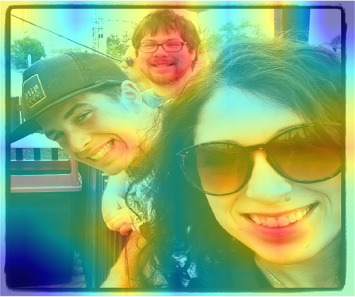	Three friends smile outdoors, capturing joy, warmth, and shared happiness in a relaxed moment together

Images reprinted with permission from “Building a Large Scale Dataset for Image Emotion Recognition: The Fine Print and The Benchmark” by Quanzeng You, Jiebo Luo, Hailin Jin, and Jianchao Yang, licensed under ©2016 Association for the Advancement of Artificial Intelligence (AAAI), [Source: AAAI Conference Proceedings].

**Table 10:** Examples of images with emotional concepts (ANPs) and sentence descriptions (Description).

**Image**	**ANPs**	**Description**
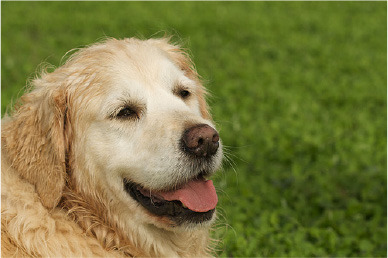	Smiling_Dog Dirty_Mouth Happy_Dog Muddy_Dog Friendly_Dog	A golden dog relaxes peacefully on soft grass, radiating calm and comfort, enjoying a carefree moment in nature.
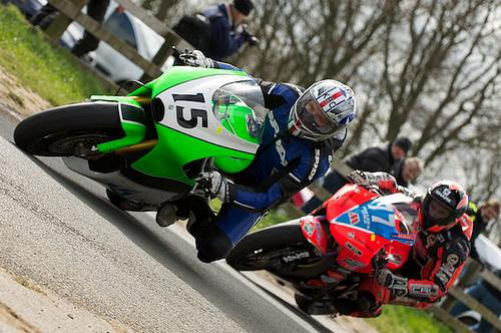	Classic_Race Tough_Race Classic_Sport Classic_Cars Young_Driver	Two focused racers lean aggressively through a sharp turn, embodying thrill, speed, and fierce determination to win.
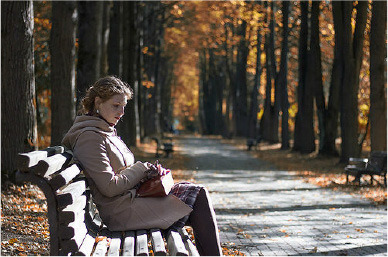	Peaceful_Park Sunny_Winter Little_Boy Playful_Kids Favorite_Dress	A solitary woman sits in an autumn park, absorbed in quiet thoughts, embodying calm introspection and gentle melancholy.

Images reprinted with permission from “Building a Large Scale Dataset for Image Emotion Recognition: The Fine Print and The Benchmark” by Quanzeng You, Jiebo Luo, Hailin Jin, and Jianchao Yang, licensed under ©2016 Association for the Advancement of Artificial Intelligence (AAAI), [Source: AAAI Conference Proceedings].

## 5 Conclusion

This paper proposed the novel OSAFN (Object-Scene Attention Fusion Network) for introducing object and scene-aware semantics as auxiliary information into image emotion classification (IEC) tasks. We leveraged emotional concepts to capture object-level affective cues and designed an Appraisal-based Chain-of-Thought (Appraisal-CoT) prompt to guide large language models in generating scene descriptions, achieving a complementary information of visual content. Two dedicated attention modules, visual object attention and visual scene attention, were used to learn fine-grained relationships between semantic and visual features. Finally, an adaptive fusion weighted the two streams, and a polarity-aware contrastive loss emphasized difficult samples while respecting the hierarchical structure of emotions. Extensive experiments on four public emotion datasets demonstrated consistent performance gains over state-of-the-art methods, validating that psychologically informed semantic assistance narrows the affective gap between pixel-level appearance and high-level emotions. One limitation is that both emotional concepts and image descriptions are ultimately text driven; they may under-represent abstract artworks or images lacking explicit semantic entities. In future work, we will explore emotion analysis for such semantics-sparse images and investigate lightweight prompting strategies to reduce LLM inference cost while preserving reasoning quality.

## Data Availability

The original contributions presented in the study are included in the article/supplementary material, further inquiries can be directed to the corresponding author/s.
